# Ocular manifestations of transthyretin-related familial amyloid polyneuropathy

**DOI:** 10.1186/1750-1172-10-S1-O19

**Published:** 2015-11-02

**Authors:** Antoine Rousseau, Emmanuel Barreau, Julia Meney, Zoia Mincheva, Cécile Cauquil, Marie Théaudin, Marc Labetoulle, David Adams

**Affiliations:** 1 French Reference Center for FAP (NNERF), Bicêtre Hospital, Ophthalmology, 94275, Le Kremlin-Bicêtre, France; 2 French Reference Center for FAP (NNERF), Bicêtre Hospital, Neurology, 94275, Le Kremlin-Bicêtre, France

## Background

Ocular manifestations of transthyretin-related familial amyloid polyneuropathy (TTR-FAP) mainly include keratoconjunctivitis sicca, secondary glaucoma and vitreous deposits. Because liver transplantation (LT) and symptomatic treatments greatly improve life expectancy of patients, ocular involvement is becoming a more frequent challenge to address. We aimed at studying the prevalence and the clinical characteristics of ocular manifestations of TTR-FAP.

## Methods

This prospective monocentric observational study was conducted at the French national reference center for TTR-FAP. Genetically confirmed TTR-FAP patients had a complete neurologic and ophthalmologic evaluation. Sensorimotor polyneuropathy (SPN) was staged with the Polyneuropathy Disability (PND) score, vegetative neuropathy was staged with the Compound Autonomic Dysfunction Test (CADT). Ophthalmological examination included best corrected visual acuity (BCVA), Schirmer test, intraocular pressure (IOP), slit lamp photographs, gonioscopy, fundus examination with retinography. Medical and surgical treatments were analyzed for all patients.

## Results

One hundred and three patients (60 males and 43 females), aged 26-83 years, (mean 55.8±14.1 years), were included. Mean delay between first symptoms and inclusion was 7.0±5.4 years. Patients of Portuguese origin accounted for 41% (N=42). Val30Met mutation was present in 69 patients (67%). Ocular Hypertension (OHT) and glaucoma occurred in 17 patients (16.5%), were associated with amyloid deposits in the anterior chamber in 76.5% of cases and were more frequent among Val30Met carriers (p<0.05). Amyloid vitreous deposits were present in 21 patients (20.3%). Lacrimal hyposecretion (Schirmer < 5 mm/5 minutes) was found in at least one eye in 38.9% of patients. A BCVA of 20/200 or worse in one eye was present in 12 patients (11.6%) and was caused by secondary glaucoma in 58.3% of cases. OHT/glaucoma and vitreous amyloid deposits were significantly more frequent in patients with autonomic neuropathy, long duration of disease (>5 years) and history of liver transplantation (p<0.05). Anterior chamber amyloid deposits were more frequent in patients with severe SPN (PND ≥ 2, p<0.05).

## Conclusions

Ocular manifestations of TTR-FAP are common. Vision-threatening manifestations of TTR-FAP are more frequent in patients with a long duration of disease and a history of liver transplantation. Secondary glaucoma is more frequent in Val30Met patients and is the main cause of severe irreversible visual impairment. Ophthalmological testing of TTR-FAP patients should be planned on a regular basis in order to detect and treat ocular manifestations as early as possible.

